# Current status and factors influencing the work readiness of middle-aged and young postoperative lung cancer patients

**DOI:** 10.1097/MD.0000000000039155

**Published:** 2024-08-02

**Authors:** Yuanyuan Yin, Xingxia Long, Jie Zhang, Mei Yang

**Affiliations:** a Department of Thoracic Surgery, West China Hospital, Sichuan University, Chengdu, China; b West China School of Nursing, Sichuan University, Chengdu, China.

**Keywords:** influencing factors, middle-aged and young, postoperative lung cancer, return-to-work readiness

## Abstract

To identify the current status of return-to-work readiness and analyze its influencing factors among middle-aged and young postoperative lung cancer patients. From July 2022 to February 2023, a total of 144 middle-aged and young postoperative lung cancer patients who had been treated in the Department of Thoracic Surgery of West China Hospital, Sichuan University and had not returned to work were selected as the research subjects. A general information questionnaire, the Readiness for Return-To-Work (RRTW) Scale, the General Self-Efficacy Scale (GSES), and the Simplified Coping Style Questionnaire (SCSQ) were used for the survey. Univariate analysis and ordinal logistic regression analysis were used to assess the current status of work readiness and its influencing factors. The distribution of work readiness from high to low was as follows: behavioral preparation-self-assessment stage, intention stage, preintention stage, and behavioral preparation-action stage. Univariate analysis showed that age, place of residence, occupation, nature of work, average family income, scope of surgery, postoperative complications, surgical site, and primary coping strategies were statistically significant (*P* < .05). The ordinal logistic regression analysis revealed that patients engaged in mentally oriented work (odds ratio [OR] = 13.78, *P* < .001), with a monthly family income of ≥ 10,000¥ (OR = 6.28, *P* = .017), proactive coping strategies (OR = 4.84, *P* = .019), and higher self-efficacy (OR = 1.17, *P* < .001) had higher work readiness. Patients engaged in other industries (OR = 0.25, *P* = .028), agricultural, forestry, and fishing labor (OR = 0.08, *P* < .001), unemployed (OR = 0.12, *P* = .038), and with a monthly family income of < 1000¥ (OR = 0.07, *P* = .026) had lower work readiness. In overall, this study suggests that the work readiness of postoperative lung cancer patients needs improvement. Occupation, nature of work, average family income, primary coping strategies, and general self-efficacy are associated with return-to-work readiness among middle-aged and young postoperative lung cancer patients.

## 1. Introduction

Lung cancer ranks as the second most common cancer worldwide and is a leading cause of cancer-related deaths.^[1]^ In China, both the incidence and mortality rates of lung cancer stand at the forefront among malignant tumors,^[2]^ posing a significant threat to human health. Clinical observations and existing research reports indicate a trend of increasing youth and lower age among lung cancer patients, with a rising number of middle-aged and young individuals diagnosed.^[3]^ Due to advancements in lung cancer screening, increased awareness of public health checkups, and the development of comprehensive treatment strategies primarily involving surgical resection, the mortality rate of lung cancer has been on the decline. However, postoperative lung cancer patients still face various physiological and psychological barriers before returning to work, such as fatigue, anxiety, and more.^[4–6]^ Studies have shown that lung cancer patients experience substantial economic pressures, with a double unemployment rate compared to the general population.^[7,8]^ Middle-aged and young patients often serve as the primary breadwinners of their families. However, the financial burden of cancer treatment, postoperative physiological and psychological symptoms, unemployment, and other issues seriously impact their personal and family life. Return-to-work readiness refers to the preparedness level for transitioning back to work after being away from work due to various events.^[9]^ Previous research has explored factors influencing the return to work among cancer survivors,^[10–12]^ yet there is a lack of studies focusing on middle-aged and young postoperative lung cancer patients.

Therefore, the current status and influencing factors of return-to-work readiness among this group deserve attention. This paper aims to provide guidance and recommendations for the development of intervention strategies aimed at assisting middle-aged and young postoperative lung cancer patients in returning to work, contributing to their recovery of work capacity.

## 2. Materials and methods

A total of 144 postoperative lung cancer patients treated in the Department of Thoracic Surgery, West China Hospital, Sichuan University from July 2022 to February 2023 were selected as the study subjects. After undergoing standardized training, three researchers distributed questionnaires through WeChat or conducted telephone follow-ups with the patients to complete the survey within 30 days after their discharge from the hospital, upon obtaining their consent. A total of 178 questionnaires were distributed, and 144 valid questionnaires were collected, resulting in an effective response rate of 80.90%.

### 2.1. Inclusion and exclusion criteria

Inclusion criteria were as follows: pathologically diagnosed with lung cancer; diagnosed while employed, currently not returned to work; surgery performed at least one month prior; aged between 18 and 60 years; adequate understanding of their own medical condition, informed consent, and voluntary participation in the study.

Exclusion criteria were as follows: severe cognitive or mental disorders; concurrent severe heart, liver, or kidney dysfunction; concomitant other malignant tumors; unwillingness to participate in the study.

### 2.2. Survey instruments

#### 2.2.1. General information questionnaire

Self-designed general information questionnaire, including demographic data such as gender, age, place of residence, number of children, education level, marital status, average monthly family income, type of medical insurance, as well as disease-related information like cancer type, surgical approach, postoperative complications, surgical site, duration of postoperative drainage, and so on.

#### 2.2.2. Readiness for Return-To Work (RRTW) Scale

The RRTW scale, developed by Canadian scholar Franche et al,^[13]^ was translated and revised for use in China by scholars including Cao et al in 2019.^[9]^ It was adapted for evaluating breast cancer patients, demonstrating a Cronbach’s α coefficient of 0.75 to 0.84 and a content validity of 0.96. The scale comprises two parts: Part 1 evaluates the readiness of patients who have not returned to work, encompassing four dimensions – preintention, intention, action readiness-self-assessment, and action readiness-behavior – with a total of 13 items. Part 2 assesses the readiness of patients who have returned to work, with a total of 9 items, covering the dimensions of uncertainty maintenance and active maintenance. A Likert 5-point scale is employed, and the scores for items within each dimension are summed to yield the dimension total score. The highest score across dimensions indicates the current readiness stage. A higher stage implies a higher level of readiness for returning to work and a more comprehensive preparation for reemployment. Given that this study focuses on young and middle-aged lung cancer patients who have not returned to work, only the first part of the scale is utilized.

#### 2.2.3. General Self Efficacy Scale (GSES)

The General Self-Efficacy Scale (GSES) developed by Schwarzer^[14]^ and translated by Wang et al,^[15]^ has been widely applied to cancer patient populations.^[16,17]^ It is used to assess individuals’ beliefs in their ability to plan and execute an action process. The scale comprises a total of 10 items, rated on a 4-point Likert scale ranging from “Completely Incorrect (1 point)” to “Completely Correct (4 points),” with a total score ranging from 10 to 40. A higher score indicates a stronger sense of self-efficacy. The Cronbach’s α coefficient for the scale ranges from 0.87 to 0.91.

#### 2.2.4. Simplified Coping Style Questionnaire (SCSQ)

The SCSQ, translated and compiled by Jie Yaning,^[18]^ consists of two dimensions: positive coping (items 1–12) and negative coping (items 13–20). It employs a 3-point Likert scale for scoring, with a total score of 60. A higher score in a particular dimension indicates a greater tendency to use that coping style. This questionnaire, which has been widely used to investigate coping styles among cancer patients, has a Cronbach’s α coefficient ranging from 0.78 to 0.90.^[18,19]^

#### 2.2.5. Statistical analysis

Data were collected using Epidata 3.1, and statistical analysis was performed using SPSS 24.0. Descriptive statistics, such as percentages and frequencies, were used to describe categorical data, while means ± standard deviations were used for continuous data. Nonparametric Mann–Whitney *U* tests were used for univariate analysis. The results of the Mann–Whitney *U* test were presented as *Z* or *H* values along with *P* values. *Z* represented the rank sum test for comparing two groups of samples, while *H* represented the rank sum test for comparing multiple groups of samples. The *Z*/*H* values indicated the standard deviation of the *U* statistic relative to the expected value. A larger *Z*/*H* value indicated a more significant difference between the 2 groups. The variables showing statistical differences will be further categorized and included in the multivariable analysis. And ordered logistic regression was used for multivariate analysis. The significance level was set at α = 0.05 for all tests.

## 3. Results

### 3.1. Current status and univariate analysis of return-to-work readiness levels in young and middle-aged postoperative lung cancer patients

A total of 144 young and middle-aged postoperative lung cancer patients were included in this study. The majority of patients were in the behavior preparation-self-assessment stage, accounting for 82 cases (56.94%). Most patients were in the intention stage, comprising 48 cases (33.33%). A small number of patients were in the preintention stage (8 cases, 5.56%) and the behavior preparation-action stage (6 cases, 4.17%). Univariate analysis revealed that age, place of residence, occupation, nature of work, average family income level, scope of surgery, postoperative complications, surgical site, and primary coping strategies were statistically significant (*P* < .05). However, gender, number of children, marital status, type of medical insurance, cancer type, cancer staging, surgical approach, and postoperative drainage time showed no statistically significant differences (*P* > .05). Detailed information was presented in Table 1.

**Table 1:** Univariate analysis of return-to-work readiness levels in young and middle-aged postoperative lung cancer patients.

Variables	Cases (%)	Preintention stage (n = 8)	Intention stage (n = 48)	Behavioral preparation – self-assessment stage (n = 82)	Behavioral preparation – action stage (n = 6)	*Z/H* value	*P* value
Gender						0.050	.823
Male	50 (34.8)	2 (4.0)	17 (34.0)	29 (58.0)	2 (4.0)		
Female	94 (65.2)	6 (6.4)	31 (33)	53 (56.4)	4 (4.2)		
Age (yr)						20.075	<.001
≤30	11 (7.6)	1 (9.1)	3 (27.3)	6 (54.5)	1 (9.1)		
31–40	41 (28.5)	1 (2.4)	6 (14.6)	33 (80.6)	1 (2.4)		
41–50	57 (39.6)	2 (3.5)	19 (33.3)	32 (56.2)	4 (7.0)		
51–60	35 (24.3)	4 (11.4)	20 (57.1)	11 (31.4)	0 (0.0)		
Residential location						11.587	.001
Urban	87 (60.4)	2 (2.3)	22 (25.3)	59 (67.8)	4 (4.6)		
Rural	57 (39.6)	6 (10.5)	26 (45.6)	23 (40.4)	2 (3.5)		
Number of children						0.677	.713
0	41 (28.5)	3 (7.3)	14 (34.2)	24 (58.5)	0 (0.0)		
1	55 (38.2)	2 (3.6)	19 (34.5)	31 (56.4)	3 (5.5)		
≥2	48 (33.3)	3 (6.3)	15 (31.2)	27 (56.2)	3 (6.3)		
Level of education						6.131	.190
Primary school and below	20 (13.9)	0 (0.0)	4 (20.0)	14 (70.0)	2 (10.0)		
Junior high school	17 (11.8)	1 (5.9)	5 (29.4)	10 (58.8)	1 (5.9)		
High school/vocational school	26 (18.0)	2 (7.7)	11 (42.3)	12 (46.2)	1 (3.8)		
College	21 (14.6)	1 (4.8)	9 (42.8)	11 (52.4)	0 (0.0)		
Bachelor’s degree or higher	60 (41.7)	4 (6.7)	19 (31.7)	35 (58.3)	2 (3.3)		
Marital status						2.786	.248
Single	19 (13.2)	2 (10.5)	4 (21.1)	13 (68.4)	0 (0.0)		
Married	100 (69.4)	4 (4.0)	40 (40.0)	52 (52.0)	4 (4.0)		
Divorced/widowed	25 (17.4)	2 (8.0)	4 (16.0)	17 (68.0)	2 (8.0)		
Occupation						47.296	<.001
Enterprise and public institution	78 (54.2)	1 (1.3)	8 (10.3)	66 (84.6)	3 (3.8)		
Agriculture, animal husbandry, fishing, and forestry industry	24 (16.7)	1 (4.2)	20 (83.3)	2 (8.3)	1 (4.2)		
Others	34 (23.6)	4 (11.8)	16 (47.0)	13 (38.2)	1 (2.9)		
Unemployed	8 (5.6)	2 (25.0)	4 (50.0)	1 (12.5)	1 (12.5)		
Nature of work						47.985	<.001
Intellectual work	66 (45.8)	0 (0.0)	6 (9.1)	54 (81.8)	6 (9.1)		
Physically demanding work	78 (54.2)	8 (10.3)	42 (53.8)	28 (35.9)	0 (0.0)		
Average monthly household income (¥)						24.369	<.001
<1000	7 (4.9)	2 (28.6)	4 (57.1)	1 (14.3)	0 (0.0)		
1000–4999	64 (44.4)	4 (6.2)	28 (43.8)	32 (50.0)	0 (0.0)		
5000–9999	55 (38.2)	2 (3.6)	15 (27.3)	35 (63.6)	3 (5.5)		
≥10,000	18 (12.5)	0 (0.0)	1 (5.5)	14 (77.8)	3 (16.7)		
Medical insurance type						5.457	.065
Self-pay	14 (9.7)	1 (7.1)	8 (57.1)	5 (35.8)	0 (0.0)		
With medical insurance	104 (72.2)	4 (3.8)	30 (28.9)	67 (64.4)	3 (2.9)		
With commercial insurance	26 (18.1)	3 (11.5)	10 (38.5)	10 (38.5)	3 (11.5)		
Cancer type						1.290	.256
Squamous cell carcinoma	44 (30.6)	4 (9.1)	9 (20.5)	29 (65.9)	2 (4.5)		
Adenocarcinoma	100 (69.4)	4 (4.0)	39 (39.0)	53 (53.0)	4 (4.0)		
TNM stage						0.532	.767
I	115 (79.9)	6 (5.2)	37 (32.2)	68 (59.1)	4 (3.5)		
II	22 (15.3)	2 (9.1)	8 (36.4)	11 (50.0)	1 (4.5)		
III	7 (4.8)	0 (0.0)	3 (42.9)	3 (42.9)	1 (14.2)		
Surgical procedure						0.710	.339
RATS	34 (23.6)	2 (5.9)	14 (41.1)	16 (47.1)	2 (5.9)		
VATS	110 (76.4)	6 (5.5)	34 (30.9)	66 (60.0)	4 (3.6)		
Surgical scope						6.051	.049
Wedge resection	82 (56.9)	1 (1.2)	24 (29.3)	54 (65.9)	3 (3.6)		
Segmentectomy	35 (24.3)	1 (2.8)	17 (48.6)	15 (42.9)	2 (5.7)		
Lobectomy	27 (18.8)	6 (22.2)	7 (25.9)	13 (48.2)	1 (3.7)		
Duration of postoperative intubation (d)						1.046	.790
1–3	77 (53.5)	3 (3.9)	26 (33.8)	45 (58.4)	3 (3.9)		
3–5	48 (33.3)	3 (6.3)	16 (33.3)	27 (56.3)	2 (4.1)		
5–7	9 (6.3)	1 (11.1)	2 (22.2)	5 (55.6)	1 (11.1)		
>7	10 (6.9)	1 (10.0)	4 (40.0)	5 (50.0)	0 (0.0)		
Postoperative complication						4.706	.030
No	137 (95.1)	6 (4.4)	45 (32.8)	80 (58.4)	6 (4.4)		
Yes	7 (4.9)	2 (28.6)	3 (42.8)	2 (28.6)	0 (0.0)		
Surgical site						6.015	.049
Left	58 (40.2)	4 (6.9)	19 (32.8)	31 (53.4)	4 (6.9)		
Right	77 (53.5)	3 (3.9)	23 (29.9)	49 (63.6)	2 (2.6)		
Bilateral	9 (6.3)	1 (11.1)	6 (66.7)	2 (22.2)	0 (0.0)		
Primary coping strategies						8.753	.003
Positive coping	116 (80.6)	4 (3.4)	35 (30.2)	71 (61.2)	6 (5.2)		
Negative coping	28 (19.4)	4 (14.3)	13 (46.4)	11 (39.3)	0 (0.0)		

RATS = robot-assisted thoracic surgery, TNM = tumor-node-metastasis, VATS = video-assisted thoracoscopic surgery.

### 3.2. Multivariate analysis of factors affecting the return-to-work readiness of young and middle-aged postoperative lung cancer patients

Ordinal logistic regression was conducted with variables that showed statistical significance in the univariate analysis (age, residence, occupation, job nature, average family income, scope of surgery, postoperative complications, surgical site, and primary coping method), along with the GSES as independent variables and work readiness as the dependent variable. The assignment of values for independent variables is detailed in Table 2. The ordered logistic regression analysis revealed that occupation, job nature, average family monthly income, primary coping method, and general self-efficacy were influencing factors of work readiness among young and middle-aged postoperative lung cancer patients. Among these, patients engaged in primarily mental work, those with an average monthly family income of ≥ 10,000¥, those adopting a proactive coping style, and those with higher levels of general self-efficacy exhibited higher work readiness (OR = 13.78, *P* < .001; OR = 6.28, *P* = .017; OR = 4.84, *P* = .019; OR = 1.17, *P* < .001). Conversely, patients in other industries, agricultural, forestry, and fishery labor, unemployed individuals, and those with an average monthly family income of < 1000¥ showed lower work readiness (OR = 0.25, *P* = .028; OR = 0.08, *P* < .001; OR = 0.12, *P* = .038; OR = 0.07, *P* = .026). Please refer to Table 3 for detailed information.

**Table 2:** Assignment of variables.

Variables	Values
Age	≤30 = 1, 31–40 = 2, 41–50 = 3, 51–60 = 4
Residential location	Urban = 1, Rural = 2
Occupation	Enterprise and public institution = 1, Agriculture, animal husbandry, fishing, and forestry industry = 2, Others = 3, Unemployed = 4
Nature of work	Intellectual work = 1, Physically demanding work = 2
Average monthly household income (¥)	<1000 = 1, 1000–4999 = 2, 5000–9999 = 3, ≥10,000 = 4
Surgical scope	Wedge resection = 1, Segmentectomy = 2, Lobectomy = 3
Postoperative complication	No = 1, Yes = 2
Surgical site	Left = 1, Right = 2, Bilateral = 3
Primary coping strategies	Positive coping = 1, Negative coping = 2
General self-efficacy	Original value input

**Table 3:** Multivariate analysis of factors affecting the return-to-work readiness of young and middle-aged postoperative lung cancer patients.

Variables	*β*	SE	Wald *x*^*2*^	*P*	OR (95% CI)
Age					
≤30	−2.046	0.96	4.547	.063	0.13 (0.02, 0.85)
31–40	1.018	0.558	3.333	.068	2.77 (0.93, 8.26)
41–50	0.627	0.522	1.439	.23	1.87 (0.67, 5.21)
51–60	Ref.	–	–	–	–
Place of residence					
Cities and towns	−0.919	0.409	5.055	.065	0.40 (0.18, 0.89)
Rural area	Ref.	–	–	–	–
Occupation					
Agriculture, animal husbandry, fishing, and forestry industry	−2.539	0.702	13.070	<.001	0.08 (0.02, 0.31)
Unemployed	−2.100	1.012	4.302	.038	0.12 (0.02, 0.89)
Others	−1.403	0.638	4.839	.028	0.25 (0.07, 0.86)
Enterprise and public institution	Ref.	–	–	–	–
Nature of work					
Intellectual work	2.623	0.652	16.213	<.001	13.78 (3.84, 49.40)
Manual labor	Ref.	–	–	–	–
Average monthly household income (¥)					
<1000	−2.598	1.169	4.938	.026	0.07 (0.01, 0.74)
≥10,000	1.837	0.768	5.725	.017	6.28 (1.39, 28.25)
1000–4999	−0.104	0.411	0.065	.799	0.90 (0.40, 2.01)
5000–9999	Ref.	–	–	–	–
Scope of operation					
Segmentectomy	−1.84	0.487	14.277	.052	0.16 (0.04, 0.41)
Lobectomy	−0.885	0.518	2.922	.087	0.41 (0.15, 1.14)
Wedge resection	Ref.	–	–	–	–
Complication					
No	0.136	0.879	0.024	.877	1.15 (0.20, 6.41)
Yes	0a	.	.	.	
Surgical site					
Bilateral	-0.504	0.913	0.304	.581	0.60 (0.10, 3.62)
Right	−0.332	0.376	0.78	.377	0.72 (0.34, 1.50)
Left	0a	.	.	.	
Primary coping strategies					
Positive coping	1.577	0.671	5.518	.019	4.84 (1.30, 18.05)
Negative coping	Ref.	–	–	–	–
General self-efficacy	0.161	0.046	12.528	<.001	1.17 (1.07, 1.29)
Stage	2.427	1.481	2.687	.101	11.32 (0.62, 206.23)
Preintention stage	2.551	1.482	2.962	.085	12.82 (0.70, 234.16)
Action preparation behavior stage	2.811	1.485	3.582	.058	16.63 (0.90, 305.82)
Action readiness self-assessment	5.448	1.541	12.504	<.001	232.30 (11.34, 4759.99)

CI = confidence interval, OR = odds ratio, SE = standard error.

## 4. Discussion

The readiness of young and middle-aged lung cancer patients after surgery to return to work in this group still needs improvement. The majority of patients were in the behavioral preparation-self-assessment stage, accounting for 82 cases (56.94%), with most of them in the intention stage, accounting for 48 cases (33.33%). A minority of patients were in the preintention stage, with only 8 cases (5.56%), and the behavioral preparation-action stage had only 6 cases (4.17%). More than half of the patients were in the self-assessment stage before returning to their jobs. This is higher than the findings reported by scholars such as Zhang Mengyao.^[11,12]^ The reason for this difference may be that, compared to lymphoma, colorectal cancer, and other cancers, most patients in this study had early-stage lung cancer, which generally leads to faster physical recovery and better prognosis. Therefore, they may have a stronger awareness of returning to work. The number of individuals in the behavioral preparation-action stage in this study was the lowest, which was lower than the findings of van Egmond and other researchers. This difference may be related to the fact that foreign cancer patients receive more professional guidance, planned vocational rehabilitation training, emotional support, and other measures before returning to work. These measures include setting up relevant counseling courses, interviews, commitment-dialogue therapy, and the use of support models, among others, to tailor personalized return-to-work plans.^[20–22]^ This suggests that future research could be based on international intervention programs and adapted to the needs and realities of young and middle-aged lung cancer patients in China, providing comprehensive services to patients and establishing a more complete follow-up system. Health departments can also provide appropriate employment and welfare policies for patients to help young and middle-aged lung cancer patients return to work.

This study reveals that occupation and job nature are influencing factors in the readiness of young and middle-aged lung cancer patients after surgery to return to work. Patients with jobs primarily involving mental activities tend to have higher levels of readiness to return to work. Research by Yang et al^[23]^ found that a job primarily involving mental activities is an important factor in patients’ readiness to return to work. Those engaged in mental work tend to have less physical activity in their daily work, which, compared to physically demanding jobs, is more conducive to their recovery from illness and a smoother return to work. Therefore, patients engaged in physically demanding work generally require longer periods of leave to facilitate their recovery, and as a result, the time required to return to work may also be longer. The study results indicate a correlation between economic income and the readiness of young and middle-aged lung cancer patients after surgery to return to work. Patients with higher average monthly family incomes tend to have higher levels of readiness to return to work, while those with significantly lower family incomes exhibit lower levels of readiness. This finding is consistent with the results of Kim et al’s research^[7]^ on lung cancer survivors, where low employment rates were associated with lower family income. The socioeconomic status of patients is an important predictive factor for returning to work, possibly due to limited access to resources and support related to work for individuals with lower incomes. However, it contradicts the results of a study by Taiwanese scholars,^[24]^ where higher monthly income groups might have better financial reserves, allowing them to take extended sick leaves.

Positive coping strategies can promote a return to work, which aligns with the findings of researchers such as Fitch and Nicoll.^[25]^ Additionally, better physical fitness can help patients adopt more positive approaches to dealing with issues arising from cancer diagnosis and treatment. Teramatsu et al^[26]^ proposed that greater lower limb strength is positively correlated with early return to work in patients after early-stage lung cancer surgery. Therefore, assessing a patient’s preoperative ability to adapt to work and implementing targeted early rehabilitation interventions are crucial. Another systematic review reported that engaging in physical exercise more than twice a week can increase the likelihood of cancer patients returning to work.^[27]^ This suggests that healthcare professionals should not only provide patients with regular postoperative rehabilitation exercises and guidance but also help them develop a correct understanding of returning to work, offer social support, and engage in comprehensive disease management for patients. This includes planning, psychological intervention, and outcome assessment, among other steps, to assist patients in adopting positive coping strategies, rebuilding their health beliefs, and actively managing their illness while regaining their social functioning, ultimately facilitating an earlier return to work.

This study demonstrates that general self-efficacy is positively correlated with the readiness to return to work. Self-efficacy is the belief in one’s ability to plan and execute an action, and it constitutes an essential component of self-concept. The results of this study indicate that higher general self-efficacy has a positive facilitating effect on returning to work. This finding aligns well with the research conducted by Wang et al,^[28]^ which analyzed 176 young and middle-aged lung cancer survivors. Wang’s study confirmed that higher self-efficacy is associated with shorter return-to-work times and job maintenance, while lower self-efficacy is significantly correlated with work withdrawal behavior. Self-efficacy plays a crucial role in sustaining motivation, promoting work engagement, and enhancing work efficiency. According to self-efficacy theory, social support can enhance self-efficacy through verbal encouragement.^[14]^ Therefore, interventions such as support groups, group cognitive-behavioral therapy, and health education for family members can be employed to increase social support for young and middle-aged lung cancer patients after surgery. This, in turn, can enhance their general self-efficacy, facilitating the restoration of their social functioning and an earlier return to work.

This study also has some limitations. Firstly, it included a relatively small number of patients and was a single-center study. Secondly, the study primarily focused on patients who had not returned to work, and did not analyze patients who had returned to work from the complete cohort undergoing the same procedures during the same period. This may introduce certain biases, and in our subsequent related research, we will further enroll and analyze patients who returned to work postoperatively.

In summary, the readiness of young and middle-aged lung cancer patients to return to work still needs improvement, and the main influencing factors include occupation, nature of work, average family monthly income, primary coping strategies, and general self-efficacy. It is recommended that healthcare professionals tailor follow-up measures to the individual circumstances of patients, considering their physical functioning, psychological state, and social aspects to enhance their readiness to return to work and facilitate their reintegration into the workforce. This can contribute to the long-term health and development of these patients. It’s worth noting that this study has certain limitations as it employed a cross-sectional research design and included a limited set of variables. Future research could consider adopting more comprehensive approaches, such as multicenter studies, prospective designs, and a combination of quantitative and qualitative methods, to further explore the current status of readiness to return to work and its influencing factors among young and middle-aged lung cancer patients after surgery.

## Author contributions

**Conceptualization:** Mei Yang.

**Data curation:** Xingxia Long, Jie Zhang, Mei Yang.

**Formal analysis:** Yuanyuan Yin, Xingxia Long, Jie Zhang.

**Investigation:** Yuanyuan Yin.

**Software:** Xingxia Long, Jie Zhang.

**Writing – original draft:** Yuanyuan Yin.

**Writing – review & editing:** Xingxia Long, Jie Zhang, Mei Yang.
